# Significance of Rosseland’s Radiative Process on Reactive Maxwell Nanofluid Flows over an Isothermally Heated Stretching Sheet in the Presence of Darcy–Forchheimer and Lorentz Forces: Towards a New Perspective on Buongiorno’s Model

**DOI:** 10.3390/mi13030368

**Published:** 2022-02-26

**Authors:** Ghulam Rasool, Anum Shafiq, Sajjad Hussain, Mostafa Zaydan, Abderrahim Wakif, Ali J. Chamkha, Muhammad Shoaib Bhutta

**Affiliations:** 1 College of International Students, Wuxi University, Wuxi 214105, China; cqu2012170@cqu.edu.cn; 2 School of Mathematics and Statistics, Nanjing University of Information Science and Technology, Nanjing 210044, China; 3 Department of Mathematics, Quaid-i-Azam University, Islamabad 44000, Pakistan; shussain@math.qau.edu.pk; 4 Laboratory of Mechanics, Faculty of Sciences Aïn Chock, Hassan II University of Casablanca, Casablanca 20000, Morocco; na.zidane@gmail.com (M.Z.); wakif.abderrahim@gmail.com (A.W.); 5 Faculty of Engineering, Kuwait College of Science and Technology, Kuwait City 35004, Kuwait; a.chamkha@kcst.edu.kw

**Keywords:** Maxwell nanofluid, Darcy–Forchheimer model, thermal radiation, chemical reaction, Brownian diffusion

## Abstract

This study aimed to investigate the consequences of the Darcy–Forchheimer medium and thermal radiation in the magnetohydrodynamic (MHD) Maxwell nanofluid flow subject to a stretching surface. The involvement of the Maxwell model provided more relaxation time to the momentum boundary layer formulation. The thermal radiation appearing from the famous Rosseland approximation was involved in the energy equation. The significant features arising from Buongiorno’s model, i.e., thermophoresis and Brownian diffusion, were retained. Governing equations, the two-dimensional partial differential equations based on symmetric components of non-Newtonian fluids in the Navier–Stokes model, were converted into one-dimensional ordinary differential equations using transformations. For fixed values of physical parameters, the solutions of the governing ODEs were obtained using the homotopy analysis method. The appearance of non-dimensional coefficients in velocity, temperature, and concentration were physical parameters. The critical parameters included thermal radiation, chemical reaction, the porosity factor, the Forchheimer number, the Deborah number, the Prandtl number, thermophoresis, and Brownian diffusion. Results were plotted in graphical form. The variation in boundary layers and corresponding profiles was discussed, followed by the concluding remarks. A comparison of the Nusselt number (heat flux rate) was also framed in graphical form for convective and non-convective/simple boundary conditions at the surface. The outcomes indicated that the thermal radiation increased the temperature profile, whereas the chemical reaction showed a reduction in the concentration profile. The drag force (skin friction) showed sufficient enhancement for the augmented values of the porosity factor. The rates of heat and mass flux also fluctuated for various values of the physical parameters. The results can help model oil reservoirs, geothermal engineering, groundwater management systems, and many others.

## 1. Introduction

The concept of nanofluid and nanotechnology in fluid flow phenomena has received enormous attraction in the community of researchers working in fluid mechanics. For sure, the thermophysical properties of fluids in the pure state have many variations, and mostly fluids face a lack of conductivity in the purely natural form. However, a mixture based on nanoparticles of some highly conductive metals in base fluids categorically reduces this deficiency, and the performance of the fluid increases drastically. Notable improvements have appeared in industry due to this innovation in nanotechnology. Specifically, the industries based on drug delivery, paints, ceramics, coatings, and similar products have been quite famous in the recent past. Similarly, enhancing the nanofluids’ heat absorption properties due to a mixture of metallic nanoparticles is another significant achievement in this regard. Nanofluids are considered top coolants in fluid flow. Therefore, the performance rate increases sufficiently. The pioneering study introducing the concept of nanofluids was reported by Choi [1], which received much attention, and after that, millions of studies have been reported so far to testify to the properties of nanofluid formulations; however, experimental evidence was provided by Buongiorno [2] in the form of the two-phase model by introducing the terms of the Brownian diffusion and thermophoresis in nanofluid flow. Benos et al. [3] reported an analytic investigation on magnetohydrodynamic naturally convective nanofluids via a horizontal cavity with local heat generation capacity. Bhattacharyya et al. [4] analyzed the impact of the hybrid structure in nanofluids and applied a statistical approach to judge the characteristics of graphene- and copper-type nanoparticles in nanofluids. Gowda et al. [5] analyzed the significance of the activation energy and second-order chemical reaction in the context of heat and mass flux rates in non-Newtonian Marangoni-driven nanofluid flow. Hussain et al. [6] disclosed the features of thermal enhancement in nanofluid flow using an embrittled cone as the core surface. Benos et al. [7] reported some crucial effects of aggregations focusing on CNT–water-based nanofluid flow subject to magnetohydrodynamic (MHD) impact. Yusuf et al. [8] considered Williamson nanofluid using an inclined surface involved with gyrotactic microorganisms to analyze the implications of MHD and bio-convection together with entropy optimization. Benos et al. [9] reported a theoretical investigation on the natural convection of CNT–water-based nanofluid flow subject to MHD under the umbrella of the revised Hamilton–Crosser theory. Apostolos et al. [10] analyzed the flow of $Al_{2}O_{3}$–water-based nanofluid considering the printed circuit heat exchangers to see the impact of the interfacial layer in the context of heat flux. Some further relevant studies can be found in [11,12,13,14] and the references cited therein.

The concept of fluid flow in a porous medium is quite natural. The flow-through a rocky surface, the natural flow of fluids through sand and dusty areas, etc., are the very realistic situations around us since the creation of the universe. In the modern world, this concept is now widely used in the manufacturing industry for multiple purposes, especially in modeling oil reservoirs, geothermal engineering, groundwater management systems, and many similar aspects [15,16,17]. Attention was received by classical Darcy law, which was valid under exceptional circumstances in situations where the porosity factor remains low. However, the classic law fails to accept the higher rate of fluid momentum through the porous medium. Thus, an improvement was needed in the classical Darcy law to enhance its applicability. Therefore, Forchheimer [18] added the squared velocity term in the momentum equation of the governing model for the classical Darcy law to tackle the higher porosity rates. Later on, Muskat [19] named this term the Forchheimer term, and the governing model was called the Darcy–Forchheimer model of fluid flow. Pal and Mondal [20] reported interesting findings on convective diffusion of the species using a non-uniform heat sink/source under the umbrella of the Darcy–Forchheimer model. Hayat et al. [21] presented the variable thermal-conductivity-based Darcy–Forchheimer flow of nanofluids using the Cattaneo–Christov model. Eid and Mabood [22] implemented the Darcy–Forchheimer model using the two-phase cross nanofluid flow. The consequence of Arrhenius activation was disclosed. Furthermore, entropy optimization was analyzed in this study. Shankaralingappa et al. [23] configured the impact of the Cattaneo–Christov theory of double diffusion on an Oldroyd-B-type fluid using a stretching surface considering the thermophoresis deposition of particles and the chemical reaction. Liquids incorporating the MHD and thermal radiation effects have been hot spots recently. The involvement of fluids with the MHD impact is relatively high in many industrial processes such as gastric medications, wound treatments, sterilized devices, medical sciences, X-ray technology, and many others. Numerous studies have been reported so far mentioning the impact of these variables in industrial applications of nanofluids. The significance of MHD is also very relatable in fluid flow analysis because it helps to control the fluid motion and thermal state of the fluid. The sudden bumps created by the magnetic field in the fluid flow phenomena are remarkably used to tackle various abnormal situations in fluid flow. Numerous related articles mention multiple parameters such as MHD, thermal radiation, chemical reaction, and many others in nanofluid flow analysis. Jamshed et al. [24] reported second-grade nanofluidic flow considering the radiation impact in a single-phase model via a flat porous surface. Sheikholeslami et al. [25] reported an analytic investigation of the MHD-type nanofluid flow subject to a semi-permeable channel. Kumar et al. [26] explored the influence of a magnetic dipole in radiative nanofluidic flow via the stretching surface using the KKL model. Kumar et al. [27] modeled Casson-type nanofluid flow using a curved stretching surface to highlight the impact of MHD and the chemical reaction. Hayat et al. [28] reported essential findings on radiative chemically reactive three-dimensional flow. Sarada et al. [29] reported the impact of magnetohydrodynamics on the heat flux rate in non-Newtonian fluids flowing over a stretching surface subject to local non-equilibrium thermal conditions. Sheikholeslami [30] analyzed the effect of thermal radiation and MHD in nanofluid flow. Charakopoulos et al. [31] examined the influence of magnetohydrodynamics in a channel flow using complex network analysis. Hamid et al. [32] reported critical data in axisymmetric nanomaterial flow towards a radiative shrinking disk. Furthermore, the impact of the Darcy–Forchheimer model, together with various parameters, including thermal radiation, chemical reaction, activation energy, and many others, were already reported (see, for example, [33,34,35,36,37,38,39]). Wakif et al. [40] reported a novel approach to the MHD analysis of Casson fluids over the horizontal surface (stretching) using the impact of thermal conductivity and temperature-dependent viscosity. Ramesh and Joshi [41] reported the MHD analysis of Jeffrey-type fluid flow between two parallel plates through a porous medium using an unsteady flow model.

The above-mentioned studies motivated the authors to look for a model comprising the given flow constraints and physical parameters. Herein, we considered the Darcy–Forchheimer flow model together with the Maxwell nanofluid boundary layer assumptions to examine the influence of thermal radiation and a porous medium using Buongiorno’s model of Brownian diffusion and thermophoresis phenomena. The governing system of equations was subject to the homotopy analysis method (HAM) (see, for example, [42,43,44,45,46]), which is a highly efficient and frequently used analytic approach to solve highly nonlinear governing equations providing the freedom of choice for choosing the linear auxiliary operators and base functions. The results were plotted graphically, and data of the skin friction and the Nusselt and Sherwood numbers are given in tables. The study concludes with a discussion on the results and concluding remarks. A comparison of results for the Nusselt number for convective boundary and the non-convective boundary is provided. The results can help model oil reservoirs, geothermal engineering, groundwater management systems, and many others.

## 2. Formulation of the Problem

**Figure 1:**
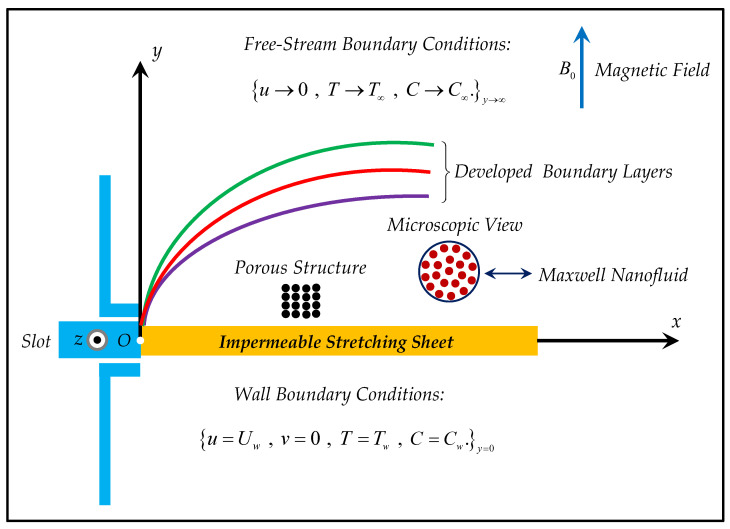
Geometry of the nanofluid flow.

Assume the two-dimensional flowchart based on a Maxwell nanofluid. The thermal radiation appearing from the famous Rosseland approximation is involved in the energy equation. In addition, the Darcy–Forchheimer model was adopted to saturate the fluid in a certain porous boundary. Furthermore, thermophoresis, Brownian diffusion, and the first-order chemical reaction were retained. The surface that generates the fluid flow was assumed to stretch linearly. Uniform magnetic impact directly influences the flow model with a term in the momentum equation. However, considering a small Reynolds number helps dismiss the magnetic field induction. The fluid was assumed to proceed alongside the x-axis, while no velocity was considered alongside the y-axis. At the initial condition, the velocity is the same as the stretching rate of the sheet, and it becomes zero as the distance approaches the free surface from the solid sheet towards the y-axis. The temperature and concentration terms are typically considered T and C, respectively having wall conditions ($T_{w},C_{w}$) at the surface and ambient conditions ($T_{\infty },C_{\infty }$) at the free surface. The physical scenario can be visualized in Figure 1. The governing equations (see, for example, [42,43,47,48]) are as follows.

(1) $$\frac{\partial u}{\partial x}+\frac{\partial v}{\partial y}=0,$$

(2) $$\begin{array}{cc} u\frac{\partial u}{\partial x}+v\frac{\partial u}{\partial y} & +\lambda_{1}\left(u^{2}\frac{\partial }{\partial x}\left(\frac{\partial u}{\partial x}\right)+v^{2}\frac{\partial }{\partial y}\left(\frac{\partial u}{\partial y}\right)+2vu\frac{\partial }{\partial x}\left(\frac{\partial u}{\partial y}\right)\right)\\ \; & =\nu \frac{\partial }{\partial y}\left(\frac{\partial u}{\partial y}\right)-\frac{\sigma B_{0}^{2}u}{\rho }-\frac{\sigma \lambda_{1}B_{0}^{2}}{\rho }\left(v\frac{\partial u}{\partial y}\right)-\left(\frac{\nu }{K_{1}}+\frac{C_{b}}{x\sqrt{K_{1}}}u\right)u, \end{array}$$

(3) $$u\frac{\partial T}{\partial x}+v\frac{\partial T}{\partial y}=\alpha \frac{\partial }{\partial y}\left(\frac{\partial T}{\partial y}\right)+\frac{\tau D_{B}}{\delta_{C}}\frac{\partial T}{\partial y}\frac{\partial C}{\partial y}+\frac{\tau D_{T}}{T_{\infty }}{\left(\frac{\partial T}{\partial y}\right)}^{2}-\frac{1}{\rho c}\frac{\partial q_{r}}{\partial y},$$

(4) $$u\frac{\partial C}{\partial x}+v\frac{\partial C}{\partial y}=D_{B}\frac{\partial }{\partial y}\left(\frac{\partial C}{\partial y}\right)+\frac{\delta_{C}D_{T}}{T_{\infty }}\frac{\partial }{\partial y}\left(\frac{\partial T}{\partial y}\right)-Cr\left(C-C_{\infty }\right).$$

(5) $$\begin{array}{c} u=U_{w}=ex,\;\;v=0,\;\;T=T_{w}\;\;C=C_{w}\;\;\mathrm{at}\;y\;=0, \end{array}$$

(6) $$\begin{array}{c} u\to 0,\;\;T\to T_{\infty },\;\;C\to C_{\infty }\;\;\mathrm{as}\;y\;\to \infty . \end{array}$$

In the last term in Equation (3), the quantity $q_{r}$ actually represents the radiative heat flux appearing from the famous Rosseland’s approximation (see, for example, [49]).

Mathematically,

(7) $$q_{r}=-\frac{4}{3}\frac{\sigma_{SB}}{k_{ABS}}\frac{\partial T^{4}}{\partial y},$$

where $\sigma_{SB}$ is called the Stefan–Boltzmann constant and $k_{ABS}$ is known as the mean absorption factor. Using the Taylor series expansion on $T^{4}$ and neglecting the second- and higher-order terms in $\left(T-T_{\infty }\right)$, one can write:

(8) $$\frac{\partial q_{r}}{\partial y}=-\frac{16}{3}\frac{\sigma_{SB}T_{\infty }^{3}}{k_{ABS}}\left(\frac{\partial^{2}T}{\partial y^{2}}\right).$$

Therefore, Equation (3) re-appears as follows:

(9) $$u\frac{\partial T}{\partial x}+v\frac{\partial T}{\partial y}=\alpha \frac{\partial }{\partial y}\left(\frac{\partial T}{\partial y}\right)+\frac{\tau D_{B}}{\delta_{C}}\frac{\partial T}{\partial y}\frac{\partial C}{\partial y}+\frac{\tau D_{T}}{T_{\infty }}{\left(\frac{\partial T}{\partial y}\right)}^{2}+\frac{16}{3(\rho c)}\frac{\sigma_{SB}T_{\infty }^{3}}{k_{ABS}}\frac{\partial^{2}T}{\partial y^{2}}.$$

Furthermore, in Equation (4), Cr represents the first-order chemical reaction. Define (see, for example, [42,50]):

(10) $$\begin{array}{cc} & u=exf^{\prime }\left(\eta \right),\;\;v=-{\left(e\nu \right)}^{1/2}f\left(\eta \right),\;\;\theta \left(\eta \right)=\frac{\left(T-T_{\infty }\right)}{\left(T_{w}-T_{\infty }\right)},\;\;\phi \left(\eta \right)=\frac{\left(C-C_{\infty }\right)}{\left(C_{w}-C_{\infty }\right)}, \end{array}$$

(11) $$\begin{array}{cc} & \eta ={\left(\frac{e}{\nu }\right)}^{1/2}y. \end{array}$$

The application of Equations (10) and (11) in Equations (1), (2), (4) and (9) results in the following non-dimensional ODEs:

(12) $$f^{\prime \prime \prime }+\left(1+M^{2}\gamma \right)ff^{\prime \prime }+2\gamma ff^{\prime }f^{\prime \prime }-\gamma f^{2}f^{\prime \prime \prime }-\left(\lambda +M^{2}\right)f^{\prime }-\left(F_{r}+1\right)f^{\prime 2}=0,$$

(13) $$\left(1+\frac{4}{3}Rd\right)\theta^{\prime \prime }+Prf\theta^{\prime }+PrN_{b}\theta^{\prime }\phi^{\prime }+PrN_{t}\theta^{\prime 2}=0,$$

(14) $$\phi^{\prime \prime }+PrLef\phi^{\prime }+\frac{N_{t}}{N_{b}}\theta^{\prime \prime }-K\phi =0,$$

(15) $$\begin{array}{c} f\left(0\right)=0,\;\;f^{\prime }\left(0\right)=1,\;\;\theta \left(0\right)=1\;\;\phi \left(0\right)=1, \end{array}$$

(16) $$\begin{array}{c} f^{\prime }\left(\infty \right)\to 0,\;\;\theta \left(\infty \right)\to 0,\;\;\phi \left(\infty \right)\to 0. \end{array}$$

In the process of non-dimensionalization, the quantities that appeared as the coefficient of f,θ,ϕ are called various physical parameters involved in the problem model. These quantities are mathematically defined as follows:

(17) $$\begin{array}{c} M^{2}=\frac{\sigma }{e}\frac{B_{0}^{2}}{\rho },\;\;\gamma =\lambda_{1}e,\;\;\lambda =\frac{\nu }{eK_{1}},\;\;K=\frac{Cr}{e}, \end{array}$$

(18) $$\begin{array}{c} F_{r}=\frac{C_{b}}{\sqrt{K_{1}}},\;\;Pr=\frac{\nu }{\alpha },\;\;Le=\frac{\alpha }{D_{B}}, \end{array}$$

(19) $$\begin{array}{c} N_{t}=\tau D_{T}\frac{\left(T_{w}-T_{\infty }\right)}{\nu T_{\infty }},N_{b}=\tau D_{B}\frac{\left(C_{w}-C_{\infty }\right)}{\nu \delta_{C}},Rd=\frac{4\sigma_{SB}T_{\infty }^{3}}{k_{f}k_{ABS}}. \end{array}$$

The quantities appearing in Equation (17) are the magnetic parameter, the Deborah number, the porosity parameter, and the first-order chemical reaction. The quantities appearing in Equation (18) are the Forchheimer number, the Prandtl number, and the Lewis factor. The first two quantities in Equation (19) are the two significant nanofluids known as thermophoresis and Brownian diffusion. The last quantity represents thermal radiation. In natural fluid flow phenomena, some critical factors affect fluid motion and the thermal state. The three crucial factors are called the drag force (skin friction), the heat flux rate (Nusselt number), and the mass flux rate (Sherwood number). The final representation of these three quantities in non-dimensional form is given below:

(20) $$\begin{array}{c} Re_{fx}^{1/2}C_{f}x=f^{\prime \prime },\;at\;\eta =0, \end{array}$$

(21) $$\begin{array}{c} Re_{x}^{-1/2}Nu_{x}=-\theta^{\prime },at\;\eta =0, \end{array}$$

(22) $$\begin{array}{c} Re_{x}^{-1/2}Sh_{x}=-\phi^{\prime },at\;\eta =0. \end{array}$$

where $Re_{x}$ is known as the local Reynolds number.

## 3. Solution Methodology

The homotopy analysis method was implemented to obtain the convergent series solutions. The graphs were prepared using Mathematica 9.0. Let,

(23) $$f_{0}=1-e^{-\eta },\;\;\theta_{0}=e^{-\eta },\;\;\phi_{0}=e^{-\eta },$$

(24) $${\hat{J}}_{f}=f^{\prime \prime \prime }-f^{\prime },\;\;{\hat{J}}_{\theta }=\theta^{\prime \prime }-\theta ,\;\;{\hat{J}}_{\phi }=\phi^{\prime \prime }-\phi ,$$

with the following hypothesis,

(25) $$\begin{array}{cc} \; & {\hat{J}}_{f}\left[L_{1}e^{-\eta }+L_{2}e^{\eta }+L_{3}\right]=0,\;\;{\hat{J}}_{\theta }\left[L_{4}e^{-\eta }+L_{5}e^{\eta }\right]=0,\;\;{\hat{J}}_{\phi }\left[L_{6}e^{-\eta }+L_{7}e^{\eta }\right]=0, \end{array}$$

where $L_{i}$, i=1,2,⋯,7, are constant numbers. Subsequently, the zeroth-order equations of deformation can be symbolized as $Q_{f}\left[\hat{f}\right]$ for the momentum equations, $Q_{\theta }\left[\hat{f},\hat{\theta },\hat{\phi }\right]$ for the energy equation, and $Q_{\phi }\left[\hat{f},\hat{\theta },\hat{\phi }\right]$ for the concentration equations given in Equations (12)–(14), such that:

(26) $$\begin{array}{cc} \; & \left(1-e\right){\hat{J}}_{f}\left[\hat{f}\left(\eta ,e\right)-f_{0}\left(\eta \right)\right]=e{\hat{h}}_{f}Q_{f}\left[\hat{f}\right],\\ \; & \left(1-e\right){\hat{J}}_{\theta }\left[\hat{\theta }\left(\eta ,e\right)-\theta_{0}\left(\eta \right)\right]=e{\hat{h}}_{\theta }Q_{\theta }\left[\hat{f},\hat{\theta },\hat{\phi }\right],\\ \; & \left(1-e\right){\hat{J}}_{\phi }\left[\hat{\phi }\left(\eta ,e\right)-\phi_{0}\left(\eta \right)\right]=e{\hat{h}}_{\phi }Q_{\phi }\left[\hat{f},\hat{\theta },\hat{\phi }\right]. \end{array}$$

with the transformed boundary conditions given in (15–16). It is important to mention here that ${\hat{h}}_{f}$ is the auxiliary function corresponding to the velocity equation, ${\hat{h}}_{\theta }$ is the auxiliary function corresponding to the energy equation, and ${\hat{h}}_{\phi }$ is the auxiliary function corresponding to the concentration equation. Furthermore, e∈[0,1] is called the embedding. $Q_{\hat{f}},Q_{\hat{\theta }}$, and $Q_{\hat{\phi }}$ are named the non-linear operators. The Taylor series implementation results in the following equations:

(27) $$\hat{f}=\sum_{i=0}^{\infty }f_{i}\left(\eta \right)e^{i},\;\;\hat{\theta }=\sum_{i=0}^{\infty }\theta_{i}\left(\eta \right)e^{i},\;\;\hat{\phi }=\sum_{i=0}^{\infty }\phi_{i}\left(\eta \right)e^{i},$$

where $E_{i}\left(\eta \right)=\frac{1}{i!}\frac{\partial^{i}E}{\partial e^{i}}{|}_{e=0}$ for $E=\hat{f},\hat{\theta }$, or $\hat{\phi }$. The efficient and smoothly convergent results are strictly dependent on the numerical choice of $\hat{h}$. The values of *e* fluctuate between e=0,1. General solutions are given as follows,

(28) $$\begin{array}{cc} \; & f_{i}=L_{1}+L_{2}e^{\eta }+L_{3}e^{-\eta }+f_{i}^{⋆}\left(\eta \right),\\ \; & \theta_{i}=L_{4}e^{\eta }+L_{5}e^{-\eta }+\theta_{i}^{⋆}\left(\eta \right),\\ \; & \phi_{i}=L_{6}e^{\eta }+L_{7}e^{-\eta }+\phi_{i}^{⋆}\left(\eta \right), \end{array}$$

where the functions with ⋆ represent the special solutions.

## 4. Results and Discussion

**Figure 2:**
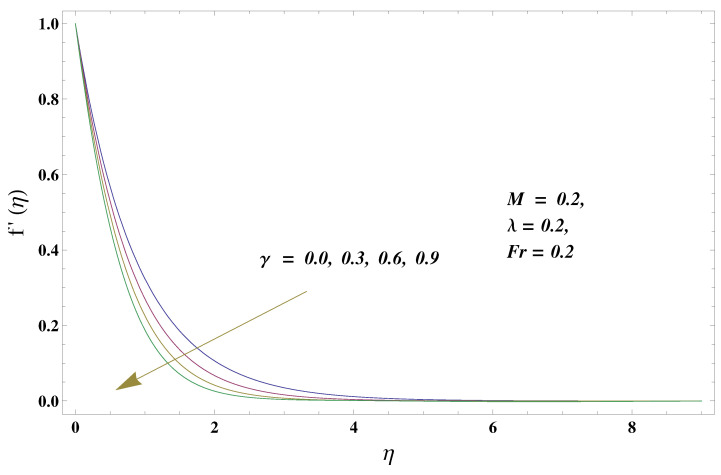
Deborah number and its impact on the velocity profile.

**Figure 3:**
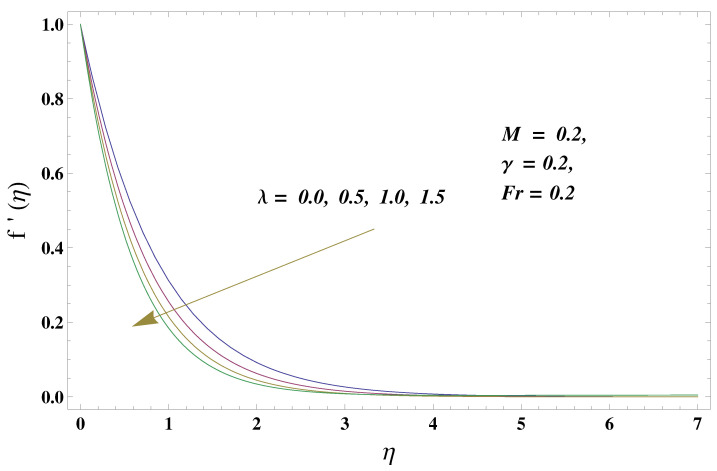
Porosity number and its impact on the velocity profile.

**Figure 4:**
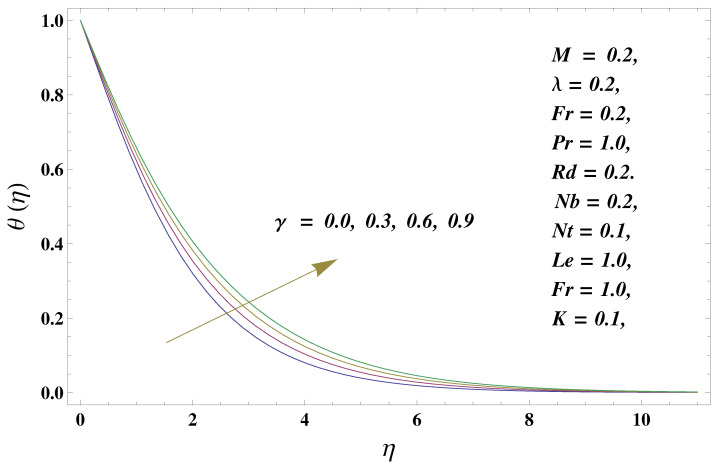
Deborah number and its impact on the temperature profile.

**Figure 5:**
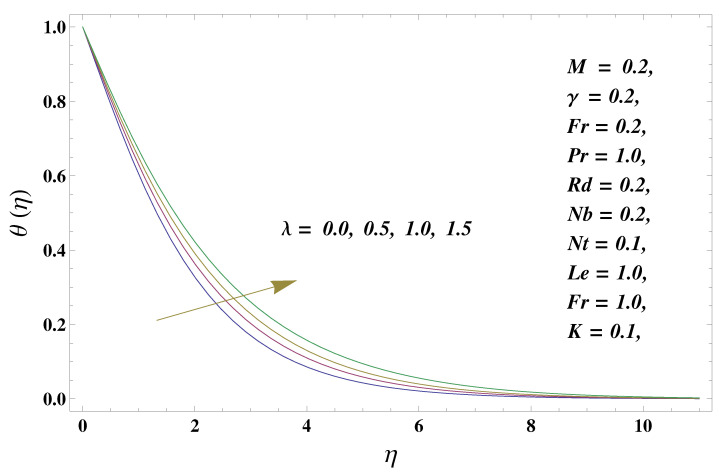
Porosity number and its impact on the velocity profile.

**Figure 6:**
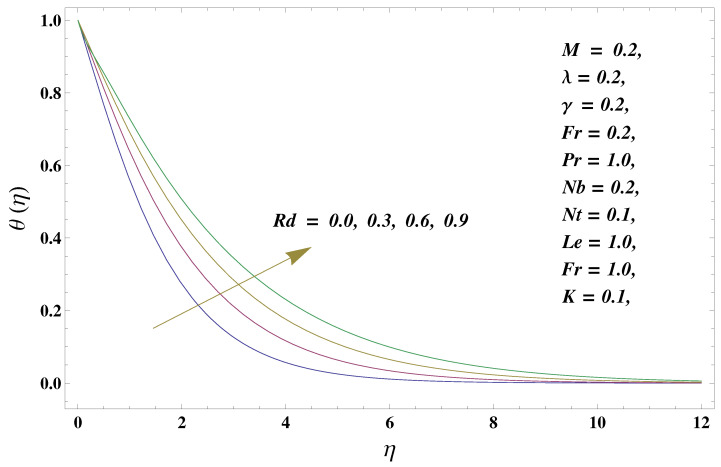
Thermal radiation and its impact on the temperature profile.

**Figure 7:**
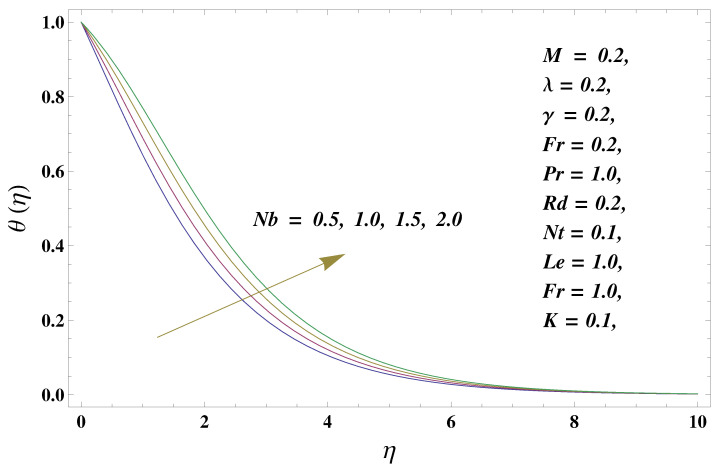
Brownian diffusion and its impact on the temperature profile.

**Figure 8:**
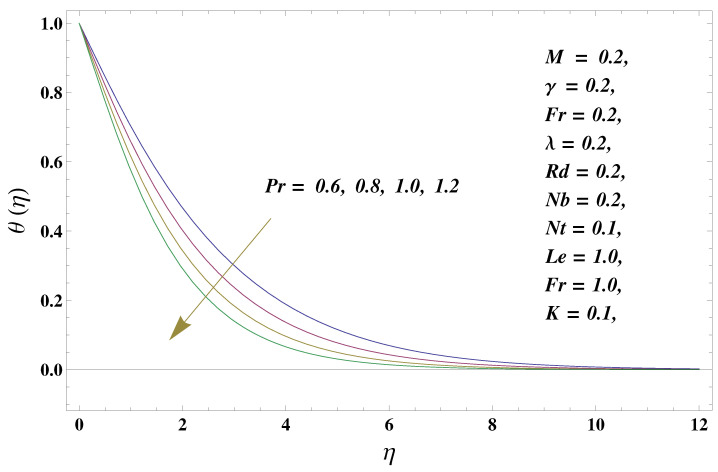
Prandtl number and its impact on the temperature profile.

**Figure 9:**
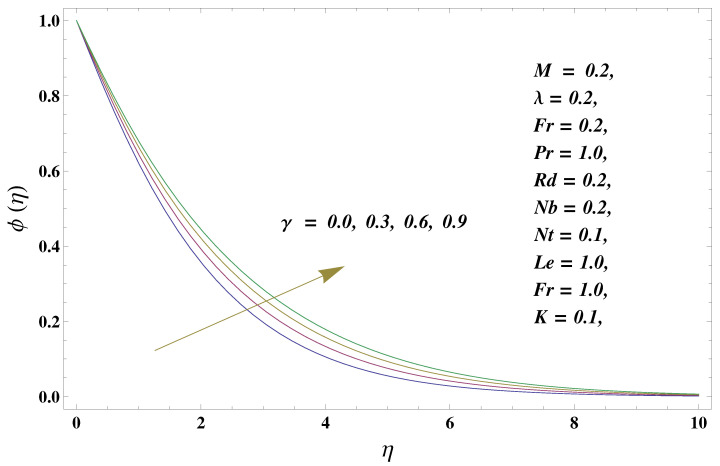
Deborah number and its impact on the concentration profile.

**Figure 10:**
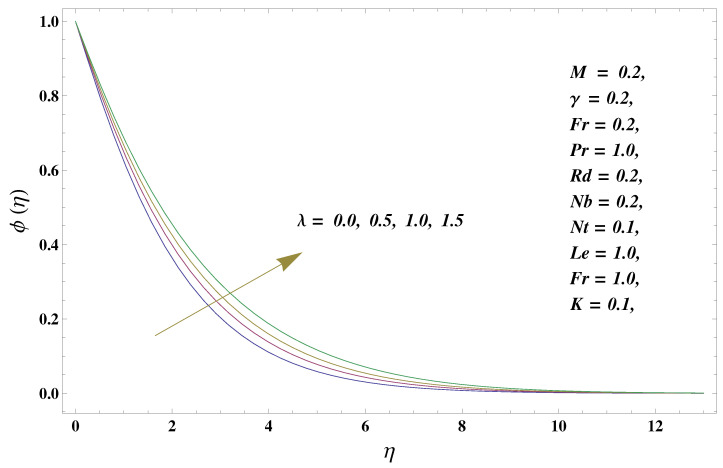
Porosity number and its impact on the concentration profile.

**Figure 11:**
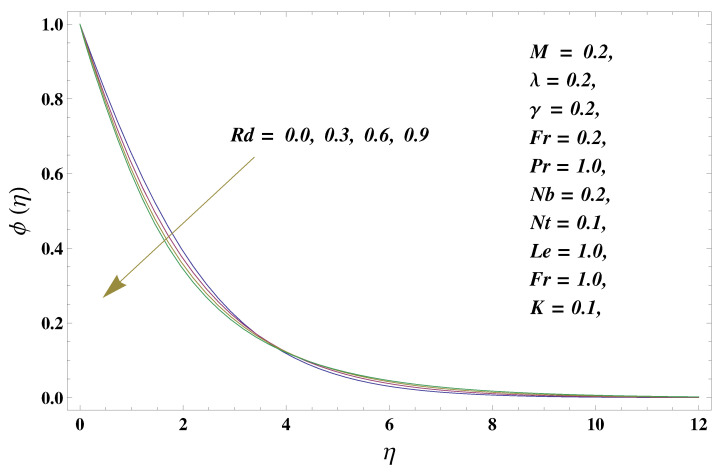
Thermal radiation and its impact on the concentration profile.

**Figure 12:**
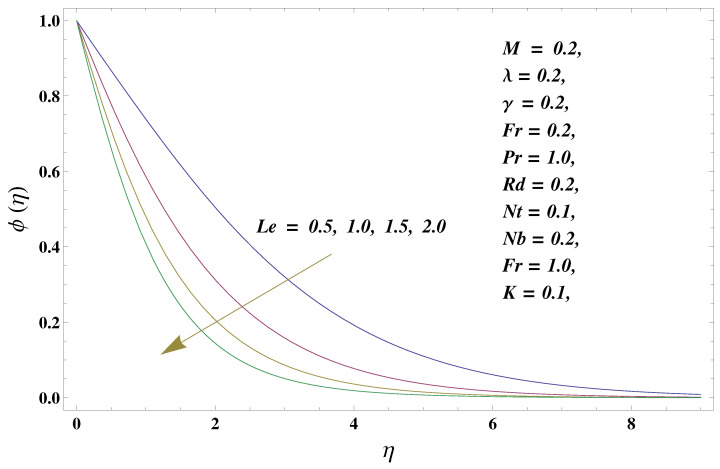
Lewis number and its impact on the concentration profile.

**Figure 13:**
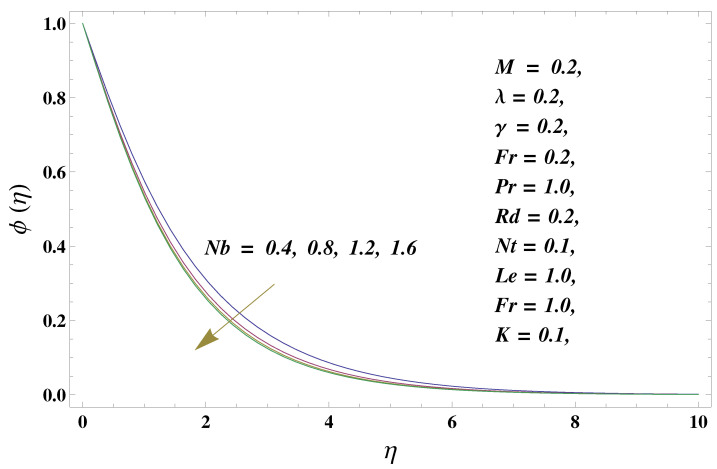
Brownian diffusion and its impact on the concentration profile.

**Figure 14:**
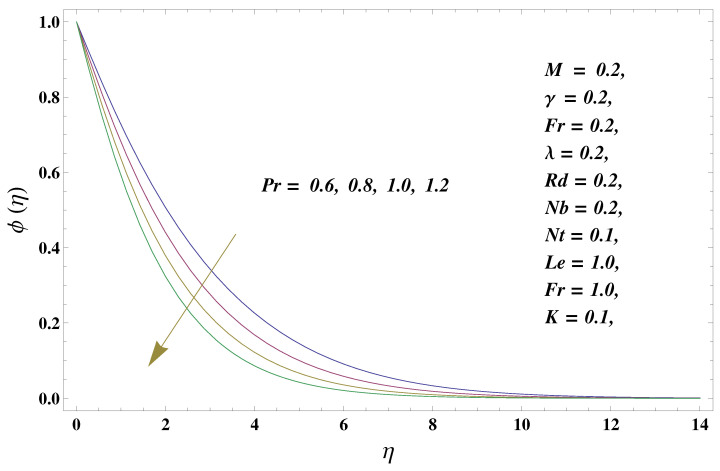
Prandtl number and its impact on the concentration profile.

**Figure 15:**
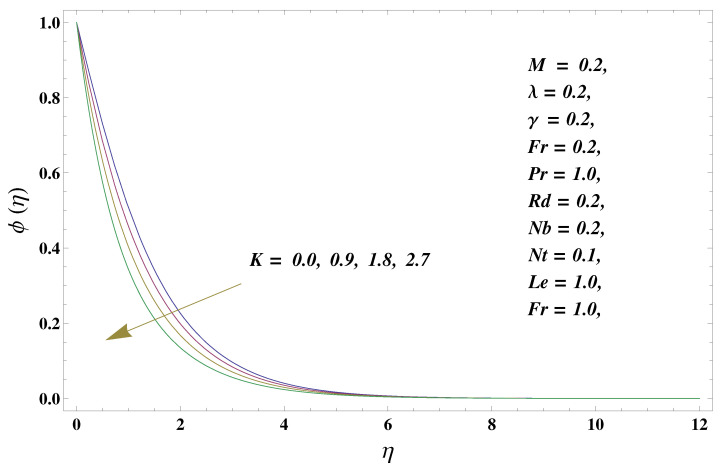
Chemical reaction and its impact on the concentration profile.

**Figure 16:**
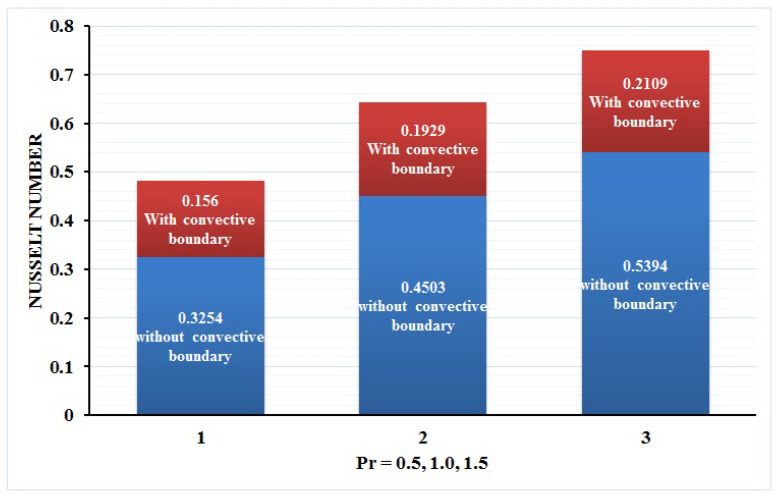
Nusselt number at Rd = 0 = K corresponding to Pr = 0.5, 1.0, 1.5.

**Figure 17:**
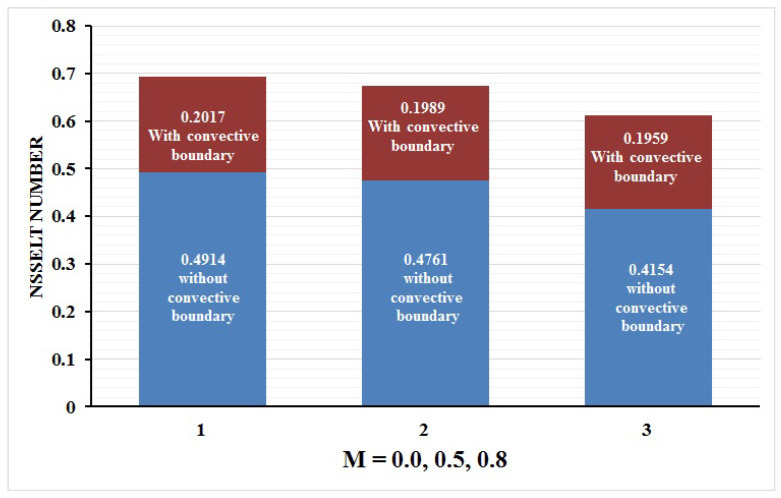
Nusselt number at Rd = 0 = K corresponding to M = 0.0, 0.5, 0.8.

In this paper, we investigated the consequences of the Darcy–Forchheimer medium and thermal radiation in the magnetohydrodynamic (MHD) Maxwell nanofluid flow subject to a stretching surface confined within the simple boundary conditions. Physical parameters such as thermal radiation, the chemical reaction, the porosity factor, the Forchheimer number, the Deborah number, the Prandtl number, thermophoresis, and Brownian diffusion and their impact on the fluid profiles are discussed in the following lines. Figure 2 and Figure 3 represent the behavior of the velocity profiles for the variation in the Deborah number and porosity factor. Specifically, Figure 2 shows the behavior of the velocity profile for altered values of the Deborah number. The constitution of the Deborah number is based on the relaxation time parameter, which in the physical context means providing more time to the nanoparticles to be diluted in the base fluid. The higher the Deborah number is, the lower the fluid velocity, and the consequent boundary layer drops to a certain extent. This appearance of the velocity profile was obtained fixing the other three physical parameters involved in the momentum equation. Figure 3 represents the variations in the velocity profile subject to the altered values of the porosity factor. Physically, the presence of the porous medium is itself a reason for the increase in the frictional retardation force offered to the fluid in motion. The higher the porous ratio in the medium, the more retardation is offered to the fluid. Therefore, the velocity profile shows a reduction in its trend for incremental values of λ. As for the energy equations, the final non-dimensional ODE involves several physical parameters already defined in the previous section. To see their impact on the temperature profile, we plotted the data in graphs given in Figure 2, Figure 3, Figure 4, Figure 5, Figure 6, Figure 7 and Figure 8. In particular, Figure 4 represents the consequent impact of the Deborah number on the temperature profile. The profile apprises the behavior of the altered, augmented values of the Deborah number. Here again, the justification of this behavior relates to the relaxation time provided to the model by the Maxwell model. The more is the relaxation time, the more is the temperature profile and vice versa. The continuous offering of more friction to the fluid in motion is the main property of the porous medium, which is mathematically involved in the model using the two important factors, the porosity factor and the Forchheimer number. The appearance of these parameters in the energy equation has a high impact on the temperature profile. Figure 5 gives this impact in the porosity factor versus the temperature profile. The higher the rate of resistance provided to the system, the higher is the system’s temperature due to the high rate of collisions between the molecules of the base fluid and the nanoparticles diluted in it. This behavior gives rise to another important aspect of fluid flow analysis, i.e., thermal radiation. The inside-out thermal radiation is another important source of raising the temperature profile. A dominant rising trend in the temperature profile due to thermal radiation can be seen in Figure 6. The result shows that even with a slight variation in the thermal radiation, a very high variation is noted in the corresponding boundary layer formulation of the temperature profile. The impact of Brownian diffusion is given in Figure 7, which is physically related to the predicted movement of nanoparticles and collisions. The higher the value of Nb, the higher is the temperature profile and vice versa. However, an inverse trend was found in the case of the Prandtl number. The higher values of the Prandtl number, as given in Figure 8, result in a reduction in the temperature profile. The constituent term of the Prandtl number involves kinematic viscosity inversely related to the thermal diffusivity. Higher values of the Prandtl number result in a reduction of thermal diffusivity and an increment in the kinematic viscosity, which results in the reduction of the temperature profile. The behavior of both the temperature and concentration profiles towards the Deborah number is quite similar. In both cases, a rise in the values of the Deborah number results in the increment of the respective profile. Figure 9 shows the same thing discussed in the above lines. Higher values of the Deborah number mean more convenience is provided to the nanoparticles to adjust and be diluted in the base fluid. The concentration profile rises after that. The porosity factor, when increased, provides more space for the nanoparticles to be spaced in the base fluid, and therefore, the consequence is shown in Figure 10. The concentration behavior in response to the altered values of thermal radiation is given in Figure 11. The interval of rising and lowering is very short. Thus, a shorter, but incremental trend is noticed for higher values of the radiation factor. The impact of Lewis’s number is quite dominant and prominent. From the Lewis number given in Equation (18), we see that the inverse relation of Brownian diffusion and thermal diffusivity is called the Lewis number. Thus, Brownian diffusion and Le are inversely proportional to each other. The higher the values of Le, the lower the diffusion will be and, therefore, the lower the concentration of nanoparticles in the base fluid, as displayed in Figure 12. Figure 13 gives the impact of Nb (Brownian motion parameter) over the concentration profile. Higher values result in a low concentration and vice versa. The Prandtl number decreases the concentration profile given in Figure 14 with the same justification as given in Figure 8 because the diffusivity is linked to the Prandtl number. The first-order chemical reaction is a source of the reduction in the concentration profile. The fluid faces a descending concentration near the surface at a lower intensity of the reactive material. However, the concentration reduces sufficiently with higher values of K, as shown in Figure 15. Table 1 provides the numerical data of the mass flux, heat flux, and wall drag (also known as skin friction) for fluctuating values of various parameters. In particular, the porosity, the Forchheimer number, and the Deborah number increase the drag force. The Nusselt number reduces for the thermal radiation factor; however, the same parameter enhances the Sherwood number. The mass flux rate is enhanced for larger values of the Lewis number. Figure 16 and Figure 17 are based on the two comparative results, i.e., with and without the convective boundary, setting the radiation factor and chemical reaction factor equal to zero. The convective boundary has a significant variation in the values of the Nusselt number as compared to a simple boundary. In both cases, the trend of the Nusselt number is the same, but the rates are different at the same values of Pr and M for both cases of different boundary conditions.

## 5. Conclusions

We considered the Darcy–Forchheimer medium and thermal radiation in the MHD Maxwell nanofluid flow subject to a stretching surface. The significant features appearing from Buongiorno’s model, i.e., thermophoresis and Brownian diffusion, were retained. The governing equations after conversion into ODEs were solved for convergent series solutions using the HAM. Graphs were plotted for the three profiles of the flow model against various physical parameters such as thermal radiation, the chemical reaction, the porosity factor, the Forchheimer number, the Deborah number, the Prandtl number, thermophoresis, and Brownian diffusion. The following salient features were the critical points in this investigation:

- The involvement of the Maxwell model in nanofluid flow provided more relaxation time for the diffusion and dilution of nanoparticles in the base fluid;
- The presence of the porosity factor was a significant source of increment in the drag force and the reduction in fluid flow along the horizontal axis;
- The impact of thermal radiation was prominent in the case of the temperature profile; however, it impacted the other two profiles as well;
- The Deborah number coming from the relaxation time provided in the Maxwell model enhanced the concentration and temperature profiles and decelerated the fluid;
- Brownian diffusion enhanced the temperature profile and reduced the concentration profile;
- The chemical reaction appeared to be a reducing factor for the concentration of nanoparticles in the base fluid;
- The trio of the porosity, the Forchheimer number, and the Deborah number increased the drag force;
- The heat flux reduced for the thermal radiation factor; however, the same parameter enhanced the mass flux rate;
- A rise was noted in the mass flux rate for augmented values of the Lewis number;
- Despite a difference in the numerical data of the Nusselt number for the convective and non-convective boundary, the trend of the increase and decrease of the flux rate was identical for both types of boundary conditions.

## Figures and Tables

**Table 1:** Numerical results of the skin friction (wall drag) and Nusselt number (heat flux). The default values are: λ=2/10, M=2/10, Fr=1, γ=2/10, K=2/10, Le=1, Nb=2/10, Nt=1/10, Pr=1, Rd=2/10.

λ	Fr	γ	*K*	Le	*M*	Nb	Nt	Pr	Rd	−Rex1/2Cfx	−Rex−1/2Nux	−Rex−1/2Shx
0.0										1.1199	0.4129	0.5306
0.3										1.2456	0.3962	0.5175
0.6										1.3601	0.3822	0.5067
	0.0									1.1786	0.5115	0.4085
	0.3									1.3065	0.4027	0.3957
	0.6									1.4242	0.3981	0.3933
		0.0								1.3514	0.4172	0.5341
		0.3								1.4492	0.3941	0.5159
		0.6								1.5233	0.3744	0.5007
			0.0							1.4235	0.4050	0.4003
			0.3							1.4235	0.3969	0.7075
			0.6							1.4235	0.3931	0.9137
				0.4						−−	0.4097	0.3353
				0.8						−−	0.4040	0.4581
				1.2						−−	0.3991	0.5846
					0.0					1.4089	0.4039	0.5236
					0.3					1.4429	0.3984	0.5193
					0.6					1.5983	0.3838	0.5079
						0.4				−−	0.3650	0.5840
						0.8				−−	0.2999	0.6142
						1.2				−−	0.2445	0.6236
							0.0			−−	0.4123	0.6312
							0.3			−−	0.3807	0.3263
							0.6			−−	0.3520	0.0868
								0.6		−−	0.3071	0.4292
								1.2		−−	0.4445	0.5693
								2.0		−−	0.5784	0.7616
									0.0	−−	0.4649	0.4990
									0.5	−−	0.3369	0.5445
									1.0	−−	0.1025	0.8359

## Data Availability

All data is available within the manuscript.

## Nomenclature

The following nomenclature is used in this manuscript:

x,y	Cartesian coordinates/*m*
u(x,y),v(x,y)	Coordinates of the velocity vector ($m\cdot s^{-1}$)
ν	Nanofluid viscosity (kinematic) ($m^{2}\cdot s^{-1}$)
μ	Nanofluid viscosity (dynamic) ($Kg\cdot m^{-1}\cdot s^{-1}$)
$C_{b}$	Inertial coefficient (*m*)
σ	Electric conductivity (${\left(\Omega \cdot m\right)}^{-1}$)
$B_{0}$	Magnetic impact ($A\cdot m^{-1}$)
$K_{1}$	Permeability ($m^{2}$)
ρ	Density of the nanofluid ($Kg\cdot m^{-3}$)
$\sigma_{SB}$	Stefan–Boltzmann constant ($W\cdot K^{-4}\cdot m^{-2}$)
α	Thermal diffusivity ($m^{2}\cdot s^{-1}$)
$k_{ABS}$	Mean absorption factor ($m^{-1}$)
*T*	Temperature (*K*)
*C*	Concentration ($mol\cdot m^{-3}$)
$T_{w}$	Temperature at the wall (*K*)
$C_{w}$	Concentration of nanoparticles at the wall ($mol\cdot m^{-3}$)
(ρc)	Nanofluid’s productive heat capacity
$T_{\infty }$	Temperature far away from the surface (boundary condition)
$C_{\infty }$	Concentration of nanoparticles far away from the surface
	(boundary condition)
Cr	Chemical reaction ($s^{-1}$)
${\left(\rho c\right)}_{p}$	Nanoparticles’ productive heat capacity ($J\cdot m^{-3}\cdot K^{-1}$)
$D_{T}$	Thermophoretic effect ($m^{2}\cdot s^{-1}$)
$D_{B}$	Brownian diffusion factor ($m^{2}\cdot s^{-1}$)
$F_{r}$	Local inertia
*M*	Magnetic parameter
*e*	Positive constant number ($s^{-1}$)
Le	Lewis factor
Nb	Brownian diffusion parameter
Pr	Prandtl factor
Nt	Thermophoretic parameter
$Nu_{x}$	Local Nusselt number (heat flux)
$Sh_{x}$	Local Sherwood number (mass flux)
$f^{\prime }$	Dimensionless velocity
η	Dimensionless variable
ϕ	Dimensionless concentration of the nanoparticles
θ	Dimensionless temperature field
$\lambda_{1}$	Relaxation time involvement
$q_{r}$	Radiative heat flux ($W\cdot m^{-2}$)
γ	Deborah number
λ	Porosity parameter
Rd	Radiation factor
*K*	First-order chemical reaction
τ	Dimensionless thermal coefficient
$\delta_{C}$	Corrective concentration coefficient
